# Age differences in the context of climate change: Does exposure to a fake consensus statement make a difference?

**DOI:** 10.1371/journal.pone.0298219

**Published:** 2024-03-13

**Authors:** Liat Ayalon

**Affiliations:** Louis and Gabi Weisfeld School of Social Work, Bar Ilan University, Ramat Gan, Israel; University of Haifa, ISRAEL

## Abstract

The present study examined whether people of different age groups respond differently to a true versus fake consensus statement concerning climate change. In total, 309 participants were randomly exposed to a true consensus statement about climate change and 311 were exposed to a false statement. Subsequently, respondents were asked to respond to items about attitudes, feelings, and behavioral intentions concerning climate change. Compared with younger people, older persons are significantly more concerned about climate change, more likely to report that climate change is real and more willing to take climate change action. Nevertheless, older persons also are more likely to be willing to post both fake and truthful information about climate change, thus, possibly serving as spreaders of both fake and truthful information. The findings suggest that it is younger people who will benefit from further education about climate change and older people who may benefit from education about the spread of information in social media. Our findings also suggest that simply providing individuals with consensus information has only limited impact on their climate change attitudes, feelings and behavioral intentions.

## Introduction

The changing climate has an omnipresent impact on the life of each and every one of us. Extremely high and low temperatures, more frequent weather events, such as droughts, floodings, hurricanes and tornadoes, and reduced biodiversity are just some of the everyday reminders of non-reversal changes brough by human (in)action [[1]. Despite these troubling changes, the engagement of citizens in pro-environmental behaviors and even more so in climate change activism has remained limited [2]. To address this limited engagement, researchers and policy stakeholders have utilized varied methods such as educational programs, intergenerational contact, and political efficacy messages among other things [3–5]. Relying on past research [6,7], this study examines the impact of factual versus fake consensus statements about climate change across different age groups.

Age is only one of numerous factors, such as gender, geographic origin, race/ethnicity, and socioeconomic status, associated with climate change inequalities [8–11]. Nonetheless, age has received considerable attention in recent climate change discourse [12]. Although both younger and older people are thought to be particularly susceptible to the negative effects of climate change, it is younger people who are expected to live longer under the influence of a dramatically changing climate [13]. Younger people also are seen as exhibiting greater concerns about the changing climate [14]. Moreover, it is younger people who are identified as the face of the climate change movement [15], whereas older people often are seen as climate deniers or greedy geezers indifferent and cynical to the negative impact of their carbon footprint. Nonetheless, it is older persons who enjoy the possession of political and economic power that the younger generations are lacking [16]. Despite these notable differences between different age groups, research concerning age differences in pro-environmental behaviors has been inconsistent, with some studies showing that older people, rather than younger people are more likely to engage in pro-environmental behaviors and other studies showing the opposite [16–18]. Nonetheless, a recent meta-analysis has shown that contrary to the popular stereotype, it is older people who are more likely to engage in pro-environmental behaviors [19].

The present study aims to examine age differences in the context of climate change through the prism of response to a scientific consensus versus fake news. One verified method to increase the public engagement in the topic of climate change is the portrayal of scientific consensus around climate change issues [6,7]. Research has demonstrated that casting doubt on the human-caused nature of climate change, increases political polarization and reduces engagement with the topic. In contrast, relaying scientific consensus statements around controversial issues, such as vaccination or climate change can sway public attitudes and willingness to act. According to the Gateway Belief Model, highlighting experts’ consensus can facilitate attitudinal changes [6,7]. Hence, change in perceived scientific consensus causes attitudinal and affective changes including support in climate action [6].

Although informative, this line of research has ignored possible age differences associated with exposure to true versus fake scientific consensus. Older people are thought to have a lesser exposure to social media and as such a more gullible approach to fake communication [20]. For instance, during the 2016 US election, older people shared the most fake information [21]. A different study that distributed political fake news articles on Facebook has found that the fake news articles had a greater likelihood to reach older people [22]. However, a different study has found no age differences in susceptibility to fake news [23], leading some to conclude that both generations lack media literacy [24].

### The present study

The study was conducted in Israel. Israel, just like the rest of the world, is impacted by the changing climate, which results in reduced biodiversity [25], forest loss [26], and significant health events [27]. Attitude-wise, however, Israeli public has shown a preference for fossil over renewable energy [28] and relatively low levels of concern about the changing climate [29]. It is important to acknowledge the fact that these attitudes do not occur in a vacuum. Current Israeli policies and regulations pay only limited attention to environmental issues [30].

Considering equivocal findings concerning the engagement of people of different age groups in climate change attitudes, feelings, and behaviors and the ability of people of different age groups to detect fake news [24,29,31], the present study examined whether people of different age groups respond differently to a true versus fake consensus statement concerning climate change. Given the substantial impact that the changing climate has on all of us, it is particularly important to identify strategies to increase public engagement with and commitment to pro-environmental behaviors and climate activism. It also is important to identify whether certain segments of the population are particularly susceptible to fake consensus statements, thus need further training and education to ensure their ability to detect fake statements and rely on accurate ones. Based on past research [32], we expected exposure to a true consensus statement to result in changes in climate change attitudes, feelings, and behavioral intentions so that a true scientific consensus would increase beliefs in climate change, worries about its effects and willingness to engage in relevant behaviors.

We also examined the following research questions:

- What age group differences in attitudes, feelings, and behavioral intentions towards climate change exist?
- How do people of different age groups respond to fake versus real consensus statements about the nature of climate change?

## Materials and methods

### Sample and procedure

The study was approved by the ethics committee of the PI’s university (# 012307, February 2023) and all respondents provided an informed consent prior to their participation. Data collection took place between 5–22 of February 2023. Respondents were recruited via an online survey agency. The sample was stratified by age (18–39, 40–64, = >65) and sex. Overall, 620 Israelis participated in the study. The decision to use these age brackets was inspired by past research which has found that on average, people roughly attributed the ending of youth to the age of 40 and the beginning of old age to the age of 64 [33]. Hence, this division results in three age groups of young adults, middle-aged adults, and older adults.

### Measures

#### Independent variables

In total, 309 respondents were randomly selected to receive the following true consensus statement about the scientific consensus concerning human involvement in the changing climate “97% of climate scientists have concluded that human-caused global warming is happening”, whereas 311 respondents received the following fake statement concerning the consensus around climate change: “over 31,000 scientists have signed a petition that there is no scientific evidence for human-caused global warming” [32,34]. The statement was provided after the demographic information was gathered but right before the dependent measures were presented. In addition, the variable age group (18–39, 40–64, > = 65) was used as an independent variable.

#### Dependent variables

Climate change attitudes, feelings, and behavioral intentions were measured following the exposure to the true/fake consensus statement. As an indicator of behavioral intention, respondents were asked about their willingness to post the true/fake consensual message, using a 1-very unlikely to 5-extremely likely scale. Next, respondents were asked to indicate their agreement or disagreement with the following attitudinal, emotional and behavioral climate change items, respectively: “climate change is a real phenomenon that impacts earth”; “I worry about the impact of climate change on my life;” “I worry about the impact of climate change on younger members of the family:” “I worry about the impact of climate change on older members of the family:” “I am willing to pay higher taxes to fight climate change.” These items were ranked on a seven-point scale, ranging from fully disagree to fully agree. Climate change items have shown evidence of face validity based on experts’ assessment and were used in past research [35,36].

#### Covariates

Sex, education (ranked on a five-point scale: primary, middle school, high school, bachelor’s degree, secondary degree), and perceived ability to detect fake news were gathered based on self-report (0-not at all; 10-full confidence).

### Analysis

All analysis were conducted using IBM SPSS Statistics version 26 [37]. First, descriptive statistics were calculated. Next, a two-way Multivariate Analysis of Covariance (MANCOVA) was conducted, with climate change attitudinal, emotional, and behavioral items as outcomes and exposure to a fake vs. a true consensus statement and age group as independent variables. The interaction between the nature of the consensus statement and age group also was examined. Covariates were included in the analysis.

## Results

### The sample

The sample was roughly equally divided across sex. There were 214 respondents under the age of 40, 159 respondents between the ages of 40 and 64 and 247 over the age of 65. Most of the sample had high school level of education or higher. On average (66/100), respondents reported moderate levels of confidence in their ability to detect fake news. The two experimental arms differed with regard to the gender distribution, with more women than men being assigned to the true consensus condition. The two arms did not differ on the other demographic variables examined in this study. See Table 1 for details.

**Table 1:** Sample characteristics (N = 620).

Variable	Total sample	True consensus	Fake consensus	p-value
	Frequency (%)/Mean(SD)	Frequency (%)/Mean(SD)	Frequency (%)/Mean(SD)	
Age				.44
<40	214(34.5%)	111 (35.9%)	103 (33.1%)	
40–64	159(25.6%)	83 (26.9%)	76 (24.4%)	
> = 65	247(39.8%)	115 (37.2%)	132 (42.4%)	
Sex				.01
Men	323(49.6%)	140(45.3%)	173(55.6%)	
Women	324(49.8%)	169(54.7%)	138(44.4%)	
Education				.12
Primary	26(4.0%)	13(4.2%)	12(3.9%)	
Middle school	20(3.1%)	8(2.6%)	9(2.9%)	
High school	263(40.4%)	133(48.0%)	121(38.6%)	
Bachelor’s degree	219(33.6%)	111(35.9%)	99(31.8%)	
Secondary degree	119(18.3%)	44(14.2%)	70(22.5%)	
Confidence in identifying fake news (0–100)	66.04(22.30)	65.80(22.09)	66.58(22.24)	.66

SD = Standard deviation.

### Age group differences and differences in the nature of the consensus statement

To test the two research questions, a two-way MANCOVA was conducted, with the independent variables being age group (18–39, 40–64, >65) and the nature of the consensus statement (true vs. false). Significant main effects were found regarding the consensus statement and regarding the age group of respondents. This indicates that there were significant differences at least on one of the items across consensus statements and across age groups, respectively. There was no significant interaction between the two, indicating that people of different age group were not likely to differentially endorse a response based on the experimental arm they were assigned to (e.g., the nature of the consensus statement). In addition, sex and education were significant covariates, indicating that some of the variations in responses can be attributed to differences in sex and education. However, perceived ability to detect fake news had no significant association with the dependent variables (e.g., climate change attitudinal, emotional, and behavioral intentions). See Table 2.

**Table 2:** Combined effects based on a two-way Multivariate-Analysis of Variance to examine the effects of age and the nature of the statement on climate change attitudes, feelings, and behavioral intentions.

Effect	Df	Pillai	F Value	P Value
Intercept	6	.51	103.59	<.001
Sex	6	.06	6.28	<.001
Education	6	.04	4.18	<.001
Confidence in identifying fake news	6	.02	1.96	.07
Consensus statement	6	.47	89.37	<.001
Age	12	.14	7.42	<.001
Consensus statement*Age	12	.02	.84	.61

df = degrees of freedom.

To examine the impact of the nature of the consensus statement on respondents’ responses, bivariate analyses are presented in Table 3. Those who were exposed to the true consensus statement were more likely to report their willingness to post the message compared with those who were exposed to the fake consensus statement. They also were more likely to indicate concerns about the impact of climate change on their lives. No other significant differences were found.

**Table 3:** Between group differences with regard to the impact of the consensus statement on climate change attitudes, feelings, and behavioral intentions.

	Total score mean(SD)	True consensus statement mean(SD)	Fake consensus statement mean(SD)	Type III SS	df	MS	F	P-value
Climate change is real (1–7)	6.16(1.20)	6.16(1.25)	6.16(1.16)	.001	1	.001	.001	.98
Worry about the effects on one’s life (1–7)	5.33(1.80)	6.35(1.06)	4.25(1.79)	680.35	1	680.35	326.92	<.001
Worry about the effects on young (1–7)	3.50(1.22)	4.92(1.76)	4.75(1.09)	5.86	1	5.86	1.89	.17
Worry about the effects on old (1–7)	4.41(1.81)	4.50(1.81)	4.33(1.80)	4.60	1	4.60	1.59	.21
Willing to post (1–5)	2.46(1.22)	2.86(1.23)	2.06(1.07)	93.69	1	93.69	71.65	<.001
Willingness to pay taxes (1–7)	4.83(1.86)	3.68(1.95)	3.80(1.95)	.07	1	.07	.02	.89

df = degree of freedom, SD = standard deviation.

Next, differences between the age groups on the dependent variables were examined. There were age group differences on all climate change items, with older people being more likely to report that climate change was real, a willingness to post the consensus message and to pay taxes and concerns about the impact of climate change on their own life as well as on younger and older family members’ lives. See Table 4. for the distribution of responses across age groups.

**Table 4:** Between group differences with regard to the impact of age group statement on climate change attitudes, feelings, and behavioral intentions.

	Total score mean(SD)	Age <40 mean(SD)	Age 40–64 mean(SD)	Age>65 mean(SD)	Type III SS	df	MS	F	Sig
Climate change is real (1–7)	6.16(1.20)	5.85(1.40)	6.20 (1.31)	6.41(.80)	28.03	2	14.02	10.50	<.001
Worry about the effects on one’s life (1–7)	5.33(1.80)	5.05(2.02)	5.25(1.82)	5.62(1.54)	44.40	2	22.20	10.67	<.001
Worry about the effects on young (1–7)	3.50(1.22)	4.22(2.04)	4.89(1.86)	5.33(1.52)	130.67	2	65.33	21.06	<.001
Worry about the effects on old (1–7)	4.41(1.81)	3.73(1.94)	4.45(1.67)	4.98(1.55)	178.72	2	89.40	30.93	<.001
Willing to post (1–5)	2.46(1.22)	2.29(1.25)	2.53(1.26)	2.56(1.19)	16.04	2	8.02	6.13	<.01
Willingness to pay taxes (1–7)	4.83(1.86)	3.07(1.93)	3.53(1.95)	4.45(1.70)	197.65	2	98.83	29.15	<.001

df = degrees of freedom, SD = standard deviation.

## Discussion

The present study examined the impact of true versus fake consensus statements on climate change attitudes, feelings, and behavioral intentions in people of different age groups. The study examined two important issues from an age-based perspective namely, climate change attitudes, feelings, and behavioral intentions and exposure to a fake consensus statement. Both topics are thought to differentially impact people of different age groups [20,38], though the exact direction of impact still remains somewhat equivocal.

The manipulation used in this study involved a random presentation of either a true or a false consensus statement, immediately followed by questions about the willingness to post the statement online as well as feelings (concerns), attitudes, and behavioral intentions about the changing climate. Our findings demonstrate that respondents were able to distinguish between a fake and a true consensus message as evident by their greater willingness to post online the true consensus statement. Those exposed to a true consensus statement also were more likely to report their concerns about the impact of climate change on their own life. Overall, these findings suggest that the manipulation was successful in creating a partial change in participants’ behavioral intentions (willingness to post the message) and climate change concerns. This is somewhat consistent with past research which has shown that although a true consensus statement resulted in a greater likelihood to estimate scientific consensus and a belief that climate change is happening, caused by humans as well as greater worries about the effects of climate change [32]. However, our findings also show that the impact of exposure to a true versus fake consensus statement was moderate at best because other climate change attitudes and behaviors assessed did not vary across the two experimental arm, thus somewhat supporting past research, which has found limited effects of fake news on people’s attitudes [39].

On the one hand, these findings are reassuring as they suggest that people possibly distinguish between true and fake climate change consensus statements (based on their lesser willingness to post fake messages), but on the other hand, the findings are somewhat disappointing as most climate change attitudes, feelings and behavioral intentions examined in this study remained unchanged following exposure to the consensus statement. This possibly suggests that people’s climate change attitudes, feelings and behavioral intentions are already quite grounded and that a simple exposure to a consensus statement regardless of its validity is of limited impact. More elaborated educational interventions are possibly needed to produce a comprehensive change. Based on the null interaction found in this study between the validity of the consensus statement and respondents’ age, we do not expect the effectiveness of such interventions to vary by age.

One finding that deserves further attention is the greater willingness of older persons to post the consensus statement regardless of its nature. This is consistent with past research which has shown that older persons are more likely to post fake information over social media [22]. Our findings suggest though, that older persons also are more likely to report willingness to post true information via social media. Hence, regardless of the validity of the information, older persons were more likely to indicate a willingness to post it. The null interaction possibly questions the claim that older persons are less likely to distinguish between fake and true information. Instead, they just tend to behave differently and be more active in posting messages compared with younger adults.

Nevertheless, age group differences in climate change attitudes, feelings and behavioral intentions were evident. Compared with younger people, older people were more likely to believe that climate change is real, to report concerns about the impact of the changing climate and to report willingness to post the consensus message about climate change and to pay higher taxes to support climate change reforms. These findings are somewhat inconsistent with past research which has found younger people to be more concerned about the changing climate and to even report ecoanxiety as a result [40]. However, it is actually older people who are more likely to engage in pro-environmental behaviors [19]. Hence, although younger people are more likely to be associated with the climate change movement [15], our findings show that compared with younger people, it is older persons who report greater concerns and are more informed and willing to act.

Despite its strength, the present study does not go without limitations. First, the sample was limited to Israel. A global study might result in differential responses based on the country of residence. This is especially needed because Israeli society appears to be less concerned about the current climate situation. It is unclear whether greater concerns about climate change would result in a differential response set to the consensus statements presented in this study. Another limitation concerns the relative limited number of items used to assess climate change attitudes, feelings, and behavioral intentions. These items were validated using face validity but are not widely used in research. In addition, although past research has shown that willingness to repost a message can serve as an indicator of the message’s credibility [41], it is also possible to claim that the association found between the true consensus statement and willingness to post the message reflects the incentive to act inherited in the message, rather than its credibility. Finally, we did not evaluate pre-test attitudes, feelings, and behavioral intentions. Although we conducted randomization, it is possible that pre-test attitudes, feelings, and behavioral intentions differed across the two experimental arms to begin with. This is particularly concerning given the dissimilarity in gender division found across the two experimental arms. However, the decision not to examine pre-test attitudes, feelings, and behavioral intentions was derived from the understanding that pretests tend to decrease the internal validity of the experiment. Instead, it has been argued that the p-value is the only valid indicator of spurious results between randomized arms [42]. It also is important to note that past research has relied on a similar method to determine the impact of a randomized intervention in the absence of pretests [43,44]. Last, the two statements used in this study do not have equal strength. The true consensus statement refers to 97% of all scientists, whereas the false statement refers to 31,000 scientists. Although these statements have been previously used [6,7], using equivalent true and false statements might have resulted in somewhat different findings.

Despite its limitations, the study addresses an important topic that has not received enough attention thus far, despite the growing impact of climate change and of fake information on our everyday life. Our findings show that compared with younger people, older persons are significantly more concerned about climate change, more likely to report that climate change is real and more willing to take climate change action. Nevertheless, older persons also are more likely to be willing to post both fake and truthful information about climate change, thus, possibly serving as spreaders of both fake and truthful information. The findings suggest that it is younger people who will benefit from further education about climate change and older people who may benefit from education about the spread of information in social media. Our findings also suggest that simply providing individuals with consensus information has only limited impact on their climate change attitudes, feelings and behavioral intentions. Thus, further educational interventions should consist of more than a consensus statement to produce a comprehensive change.
